# ShopSmart 4 Health – Protocol of a skills-based randomised controlled trial promoting fruit and vegetable consumption among socioeconomically disadvantaged women

**DOI:** 10.1186/1471-2458-13-466

**Published:** 2013-05-14

**Authors:** Kylie Ball, Sarah A McNaughton, Ha Le, Nick Andrianopoulos, Victoria Inglis, Briohny McNeilly, Irene Lichomets, Alba Granados, David Crawford

**Affiliations:** 1 Centre for Physical Activity and Nutrition Research, Deakin University, Burwood Hwy, Burwood, Victoria, 3125, Australia; 2 Deakin Health Economics, Deakin University, Burwood Hwy, Burwood, Victoria, 3125, Australia; 3 Murray Goulbourn Co-operative Co. Ltd., Victoria, Parkville, 3052, Australia

**Keywords:** Nutrition intervention, Randomised controlled trial, Socioeconomic disadvantage

## Abstract

**Background:**

There is a need for evidence on the most effective and cost-effective approaches for promoting healthy eating among groups that do not meet dietary recommendations for good health, such as those with low incomes or experiencing socioeconomic disadvantage. This paper describes the ShopSmart 4 Health study, a randomised controlled trial conducted by Deakin University, Coles Supermarkets and the Heart Foundation, to investigate the effectiveness and cost-effectiveness of a skill-building intervention for promoting increased purchasing and consumption of fruits and vegetables amongst women of low socioeconomic position (SEP).

**Methods/design:**

ShopSmart 4 Health employed a randomised controlled trial design. Women aged 18–60 years, holding a Coles store loyalty card, who shopped at Coles stores within socioeconomically disadvantaged neighbourhoods and met low-income eligibility criteria were invited to participate. Consenting women completed a baseline survey assessing food shopping and eating habits and food-related behaviours and attitudes. On receipt of their completed survey, women were randomised to either a skill-building intervention or a wait-list control condition. Intervention effects will be evaluated via self-completion surveys and using supermarket transaction sales data, collected at pre- and post-intervention and 6-month follow-up. An economic evaluation from a societal perspective using a cost-consequences approach will compare the costs and outcomes between intervention and control groups. Process evaluation will be undertaken to identify perceived value and effects of intervention components.

**Discussion:**

This study will provide data to address the currently limited evidence base regarding the effectiveness and cost-effectiveness of skill-building intervention strategies aimed at increasing fruit and vegetable consumption among socioeconomically disadvantaged women, a target group at high risk of poor diets.

**Trial registration:**

Current Controlled Trials ISRCTN48771770

## Background

Many Australians do not consume adequate quantities of foods that are important for leading healthy lives. For instance, more than 90% of Australian adults do not eat enough vegetables, and more than 48% do not eat enough fruit for good health [1]. Compared with those of high socioeconomic position (SEP), individuals of low SEP (e.g., with low levels of education, low incomes, or living in socioeconomically disadvantaged neighbourhoods) are less likely to consume amounts of fruits and vegetables [2,3]. This puts people of low SEP at increased risk of a range of serious health conditions including obesity, heart disease and stroke, and certain cancers [4].

Currently little is known about how best to promote fruit and vegetable consumption amongst persons of low SEP. Observational studies of the determinants of unhealthy eating have suggested a number of barriers to healthy eating amongst individuals of low SEP, including relatively lower levels of nutrition knowledge, lack of meal planning/preparation/cooking confidence and skills, and perceived high financial costs of healthy eating [5,6]. We previously published a multilevel study to explore intrapersonal, social and environmental factors that might explain socioeconomic variations in women’s fruit and vegetable intakes [2]. That study showed that lower intakes of fruits and vegetables amongst women of low SEP were not attributable to poorer access to stores in the local neighbourhood, but rather to intrapersonal factors, particularly less nutrition knowledge, and less consideration of health issues when purchasing foods. Other research has demonstrated that the use of strategies such as meal planning in advance and preparing a shopping list were associated with greater intakes of fruits and vegetables, suggesting that behavioural strategies may be important targets for healthy eating interventions [7]. In the global context of rising food prices, strategies focused on effective food budgeting on a restricted income may also be particularly important in supporting persons experiencing socioeconomic disadvantage to eat more healthfully.

There remains little evidence from intervention studies about the most effective means of promoting healthy eating behaviours in persons of low SEP. Three literature reviews have suggested that behavioural interventions show promise in promoting increased fruit and vegetable consumption in the general population [8-10]. Elements of effective behaviour change interventions identified in those reviews include goal-setting; recipe provision; and print materials. However, the reviews also identified a number of key gaps in the evidence. Firstly, evidence for the effectiveness of these strategies for promoting increased fruit and vegetable consumption amongst low-income or minority groups was very limited. For example, Ammerman et al. noted that ‘a serious deficit still exists in good quality published research designed to determine the relative efficacy of different intervention approaches in these high risk populations for whom conventional intervention approaches may be a poor fit with their needs’ [8]. Secondly, intervention evidence was limited by a heavy reliance on self-report measures to evaluate intervention effects. Thirdly, the reviews highlighted the lack of data on the cost-effectiveness of dietary interventions for promoting fruit and vegetable consumption. In the context of increasingly limited resources for public health, it is critical to establish whether interventions represent good ‘value-for-money’. Finally, few studies cited in the reviews reported on the mediators or mechanisms underlying any increases in fruit and vegetable consumption resulting from interventions. An understanding of these mediators is important for highlighting the most successful intervention elements and how they operate to change behaviour.

In summary, there is a need to develop and evaluate healthy eating interventions specifically addressing the needs and barriers faced by socioeconomically disadvantaged individuals; to evaluate these using objective measures; to report on intervention cost-effectiveness; and to identify the mediators of intervention effects. This paper reports on the protocol and methods of the ShopSmart 4 Health study, a randomised controlled trial designed to test the effectiveness and cost-effectiveness of a skill-building intervention promoting fruit and vegetable purchasing and consumption amongst socioeconomically disadvantaged women. Secondary aims were to test the impact of the intervention on self-efficacy for, perceived barriers to, and perceived affordability of, consuming fruits and vegetables; and to examine the contribution of self-efficacy, perceived barriers and perceived affordability as mediators of change in fruit and vegetable purchasing and consumption behaviours resulting from the intervention.

## Methods

### Study design, target group and setting

**Figure 1:**
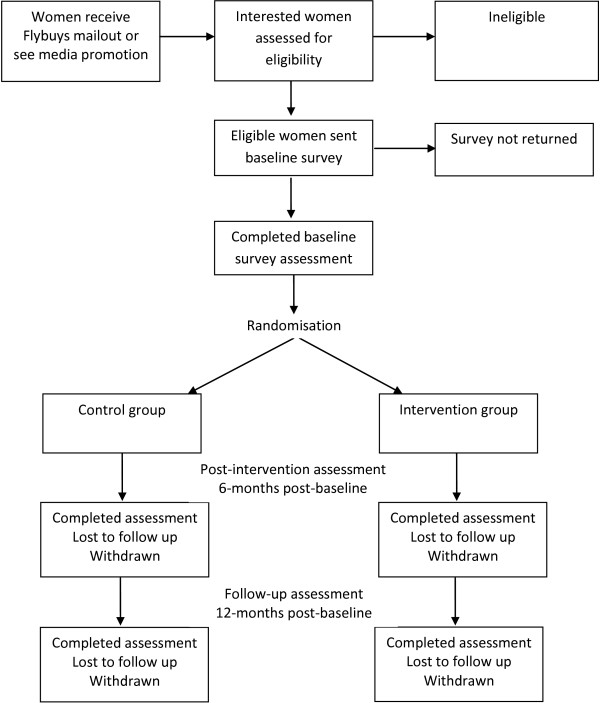
Flow chart showing participant recruitment, randomisation and evaluation of the ShopSmart intervention.

The ShopSmart 4 Health (referred to henceforth as ShopSmart) study is a randomised controlled trial with a 6-month intervention and pre-, post- and 6-month follow-up assessments of intervention effects, as outlined in Figure 1. The Deakin University Faculty of Health Human Ethics Advisory Group approved the study (HEAG-H 188/09), and it was funded by the Australian Research Council Linkage Scheme (LP0990129). The trial is registered with the International Standard Randomised Controlled Trial Number Register (ISRCTN48771770). ShopSmart focuses on women of low SEP who shop at Coles supermarkets in socioeconomically disadvantaged suburbs of Melbourne, Australia. Melbourne is the capital of the state of Victoria, and with a population of approximately 4 million people in the greater geographical area, is the second-most populous city in Australia [11]. Coles Supermarkets are the second largest grocery chain in Australia, with around 740 stores nationally. Women were the focus, since they are most often responsible for food purchasing and preparation, particularly in family households [12,13]; they thus often influence the amount and type of food eaten by other family members. A large proportion of groceries are purchased in supermarkets. For example, Australians spend over $80 billion on supermarket and grocery shopping, which accounts for 62% of the retail food sector [14]. However, there are very few supermarket-based eating interventions reported internationally, suggesting a potentially valuable missed opportunity to promote healthy eating. This study contributes to addressing this gap, building on a partnership with Coles supermarkets and the National Heart Foundation of Australia.

### Recruitment source

Participants were recruited from two catchment areas selected randomly from all neighbourhoods that were within 25km of Deakin University in Melbourne, Australia; were socioeconomically disadvantaged (i.e. being in the lowest quartile of disadvantage according to the Australian Bureau of Statistics Socioeconomic Index for Areas [15]; and that had a Coles supermarket within the neighbourhood.

### Participant identification and recruitment procedure

Coles Supermarkets have a store loyalty program called FlyBuys, which is Australia’s largest shopping rewards program, with more than 10 million cardholders nationally, and more than 60% of Australian households being members (https://www.flybuys.com.au/export/sites/default/flybuys/content/information/mediacentre/FlyBuysFastFacts.pdf, retrieved 18 November 2012). Shoppers who sign up to FlyBuys are given a credit card style membership card which can be scanned every time a purchase above five Australian dollars is made at a participating FlyBuys business. This allows members to collect points which can then be exchanged for rewards. For this study, a sample of FlyBuys members who were women, aged 18–60 years and shopped at least once every two weeks in any Coles store in either one of the catchment areas was extracted using unidentified sales data from Coles and FlyBuys. A recruitment brochure was then mailed out to the women identified, explaining the study and asking them, if interested, to complete the eligibility questions and return the brochure to the researchers. In addition, a media promotion advertising the study was undertaken in the study neighbourhoods to coincide with the mailout, and attract additional interest. Respondents to either the mailout or media promotion who met all the following criteria were eligible and were subsequently enrolled into the study: woman, aged between 18 and 60 years; the main household shopper; shops regularly (at least once every two weeks) at Coles supermarkets in one of the two defined catchment areas; a FlyBuys (Coles store loyalty card) member, or willing to sign up to FlyBuys, and willing to use their FlyBuys customer loyalty card at Coles supermarkets for the next 12 months; willing to complete three surveys across the study period, and to have purchase data collected and analysed; the only woman in the household taking part in the study; able to speak, read and write in English, and to provide informed consent; and meeting income criteria. The income criteria were carefully selected in order to recruit the target sample of socioeconomically disadvantaged women, without the need for asking potential participants sensitive income questions upon initial registration of interest. For this purpose, women were simply asked if they met ANY of the following criteria (they were not required to specify which): had a household income below AUS$1000 per week after tax; or were the holder of a health care card (a Government card available to low-income earners or welfare beneficiaries); or their main income was derived from a pension or welfare benefit. The household income cutpoint was chosen to correspond to slightly under the median after- tax household income (ignoring household composition); reported as $1183/week in August 2010 (ABS, see http://www.ausstats.abs.gov.au/ausstats/subscriber.nsf/0/DBE855896D8CA36DCA2578FB0018533C/$File/65230_2009-10.pdf). Participants gave their informed consent and all participants, whether intervention or control group, were provided with compensation to the total value of approximately $80 ($20 for each survey completed; 1000 FlyBuys bonus points, worth approximately $15; and small gifts such as pens and water bottles) for their time and commitment to the study.

### Intervention

The intervention was guided by the social cognitive theoretical model [16], which proposes that individuals adopt new behaviours through social learning, either through imitation of others, or through media sources. In addition, the intervention draws on empirical evidence of the key determinants of eating behaviours in socioeconomically disadvantaged groups, including past work suggesting the importance of addressing nutrition knowledge, budgeting and food planning and preparation skills, and perceived cost barriers [2,5-7].

An intervention mapping approach was undertaken [17] to ensure that the intervention was underpinned by a strong theoretical, empirical, and practical foundation. A literature search was conducted to identify existing materials that target increasing fruit and vegetable consumption in socioeconomically disadvantaged groups, and that addressed the key determinants and constructs of social cognitive theory. New materials were developed where there was insufficient or inadequate existing content to address the study aims. The findings of the search and mapping process suggested that selected printed materials, in conjunction with a face-to-face component, both focused on building skills, on budgeting and value for money, were promising strategies. A set of eight educational and skill-building newsletter and behaviour change resource packages, as well as a supermarket tour to complement and provide practical demonstrations of the printed information, were then developed by Accredited Practising Dietitians in collaboration with study investigators. The approach and materials shared some similarities to those adopted in a sister study, SHELf, conducted by the investigators [18]; however, ShopSmart was specifically targeted to addressing the needs of women of low SEP, and hence materials were targeted accordingly, as follows.

The materials targeted the proposed theoretical mediators, nutrition self-efficacy, perceived affordability and other perceived barriers to fruit and vegetable consumption. Intervention elements included improving nutrition knowledge and confidence, including increasing awareness of the importance of fruit and vegetables to health, highlighting the range of low-cost fruits and vegetables available and their uses, and the relative costs compared with other foods and in-season produce. The value of more affordable options (such as frozen or tinned fruits/vegetables) was also a strong focus. Women were encouraged to set specific goals in order to increase their own and their families’ vegetable consumption to meet the guidelines of 5 serves per day [19]. They were provided with goal-setting exercises to record these goals, and were reminded in each resource package to revisit them, and provided with feedback/suggestions for progressing. Skill-building activities were designed to foster behavioural skills in budgeting; meal planning; label reading and food selection in the supermarket; meal preparation strategies including preparing shopping lists prior to getting to the supermarket; and food safety and long-term storage. The packs also included advice on overcoming commonly-reported barriers to increasing fruit and vegetable consumption, such as replacing more expensive fresh produce with frozen/tinned; and involving children in choosing/shopping for/preparing meals.

### Skill-building resource packs

The eight editions of the printed newsletters and resource packs were distributed to intervention participants, with a pack sent every two weeks for the first two months and monthly for the remaining four months of the intervention. The emphasis of these newsletters was on budgeting, goal setting; meal planning; cutting costs; taste and increasing confidence and family involvement in fruit and vegetable preparation and consumption. One key source of newsletter content was FoodCent$ [20], an innovative intervention approach aimed primarily at increasing food budgeting skills to support people with limited budgets to allocate money to healthier foods. While the FoodCent$ trial showed positive changes in cooking, shopping and eating behaviours, it had not been evaluated in a randomised controlled trial. In the present study, newsletters incorporated elements of FoodCent$, including resources for participants to develop budgeting and food shopping skills.

Each newsletter also included two recipes for healthy, inexpensive meals incorporating fruits and vegetables, with cost and nutritional information provided. Resource packs also included supplementary skill-building materials (goal-setting activities, menu planners, shopping lists, seasonality and food preparation and storage guides, self-monitoring exercises) as well as links to useful websites. There was a strong focus in all resources on the specific needs of women of low SEP, particularly affordability and nutrition-related skills. The packs were reviewed by experts in the field before being pilot tested in a convenience sample of 34 women. Following pilot-testing, minor revisions were made to improve readability, interest, tone and layout.

### Supermarket tours

Supermarket tour content was developed by Accredited Practising Dietitians, and focused on reinforcing the materials provided in resource packs, as well as on providing practical, hands-on skills for food selection and preparation. Topics included health benefits; seasonality and selection of fruit and vegetables; tinned/frozen fruit and vegetable options; label reading; appropriate portion sizes; cost saving; and practical strategies for increasing fruit and vegetables in everyday meals. The emphasis was on affordability, convenience/time-saving, taste and increasing confidence/ self-efficacy. Each supermarket tour ran for approximately one hour, and was conducted by one Accredited Practising Dietitian assisted by one other member of the research staff. The tours were attended by small groups (n=1-6) of participants and held in Coles supermarkets in the study areas at times convenient to participants.

### Control group

Participants in the control group completed the assessments only, until the intervention and 6-month follow-up were complete, at which point they were offered all printed intervention materials in a single pack.

### Measures

Outcome data were collected from both intervention and control participants pre- and immediately post intervention and at 6 months following the completion of the intervention. Economic data were collected alongside the outcome data from Coles electronic sales data and participants’ self-reported postal questionnaires. Additional data on the resources used to provide the intervention were collected from records kept by project staff. Process evaluation data were collected from intervention participants immediately post-intervention and also after the supermarket tour for those completing this activity.

### Outcome variables

The primary outcome variables are vegetable purchasing and consumption. Measures of food purchasing were obtained using electronic sales data provided by Coles for consenting participants via their Flybuys cards, which participants were asked to scan each time they shopped at Coles. Individualized electronic purchasing data for each participant were collected for a 3-month period prior to intervention (retrospectively following consent), and continually during the 6-month intervention and the 6-month follow-up. Participants’ purchasing data, identified by their unique Flybuys card number, were provided in tab-delimited files and uploaded to a SQL database. The files included a unique product code; product descriptor; amount/quantity purchased; and the related expenses. For each participant, all fresh, frozen, dried and tinned vegetable items purchased were identified by product codes and the quantities (by weight) and the total expenses per each item ($).

All other primary and secondary outcomes were assessed by self-report surveys. Vegetable consumption was assessed via two measures. Firstly, respondents were asked ‘About how many serves of vegetables do you usually eat per day? (1 serve = ½ cup cooked vegetables or 1 cup salad vegetables)’ Eight response options ranged from I don’t eat vegetables, to 6 or more serves per day. This item was adapted from the 1995 National Nutrition Survey [21], where it was shown to adequately discriminate between groups with different vegetable intakes assessed by 24-hour recall. Secondly, respondents were administered a Food Frequency Questionnaire [21], including 21 items assessing the average consumption of commonly-consumed vegetables in the past 6 months, with nine response options ranging from never or less than once/month, to 6 or more times per day.

Secondary outcomes are fruit purchasing and consumption, assessed in the same way as vegetable purchasing/consumption. Frequency of consumption of energy-dense, nutrient poor foods and beverages were measured by the survey as a marker of unintended consequences (for example, increases in intakes of confectionery or sugar-sweetened beverages). Food security was assessed as a potential consequence of the intervention (‘In the last 12 months, were there any times that you ran out of food, and couldn’t afford to buy more?’) [21]. The survey also assessed theoretical mediators and behaviours and attitudes targeted by the intervention to promote these: self-efficacy for increasing fruit and vegetable consumption; perceived affordability of, and barriers to, fruit and vegetable consumption; cooking confidence; behavioural strategies related to food planning/shopping; and considerations when grocery shopping. Finally, the following sociodemographic characteristics were assessed: age, country of birth, relationships status, highest qualification (own & partner’s if applicable), employment status (own & partner’s), income category (own & household) and number of dependents; and number of adults and children for whom groceries were bought. Other covariates assessed by survey included height/weight, dieting for weight control, vegetarianism, and pregnancy.

### Economic evaluation

A cost-consequences analysis comparing the incremental costs and outcomes of the skill-building intervention to the control group will be conducted under a broad societal perspective. Key costs for the economic evaluation include program costs (dietitian and other researcher time in relation to the intervention, intervention material costs and travel expenses) and family costs (family time spent on the intervention and related travel costs, plus expenditure on fruit and vegetable consumption). The possible future effects of this nutrition education program and the related costs such as the reduced healthcare costs, the improved quality of life and the increased workforce participation are beyond the extent of the economic evaluation of this study and therefore will not be considered in the economic analysis. All costs and outcomes are expressed in their natural units. Participants will be asked where and how often they do grocery or food shopping, the amount of fruit and vegetables are bought from Coles, the average time spent food shopping at Coles and the mode of transportation as well as the travel time to get to Coles stores from home. Measured resource use will be valued using the cost of each unit of resource use such as Coles electronics sales data for household expenditure on fruit and vegetables, Australian Bureau of Statistics estimates of average Australian earnings for family time costs, Royal Automobile Club of Victoria (RACV) estimates of travel costs, etc. Extensive sensitivity analyses will be conducted on key costs and outcomes. No discount factor is applied for costs and outcomes in this less than one year trial.

Process evaluation will focus on intervention participants’ experience of all aspects of intervention implementation.

### Sample size and justification

Sample size calculations were based on the ability to detect increases in vegetable consumption of at least 0.5 serves per day (in Australia, a standard serve is equivalent to 75 grams vegetables or 150 grams fruit). There is evidence that this increase is feasible; for example, a review of the literature on fruit and vegetable intervention studies^7^ found an average increase of 0.6 serves of fruit and vegetables per day across studies. Even small population increases in fruit and vegetable consumption such as this are meaningful. For example, an increase of 80 g per day of fruit and vegetables has been estimated to reduce the risk of ischaemic heart disease by 10%, ischaemic stroke by 6%, lung cancer by 4% and oesophageal cancer by 6% [22].

Of the outcome variables (fruit and vegetable consumption), it was estimated that the most challenging behaviour to shift would be vegetable consumption, therefore sample size estimates have been based on this outcome. At the time of study design, there were no recent national nutrition data in Australia (with the last national survey conducted over 12 years ago), so these estimates were based on more recent data collected in a large community-based survey of over 1500 women [2]. In that study, women of low SEP reported mean intakes of 1.9 serves of vegetables per day (0.5 serves fewer than those of higher position), with a standard deviation of 1.1. Therefore, to detect an increase of 0.5 serves of vegetables, using the following formula to calculate the sample size for a continuous measure:

N per group = 2 * SD2 * (Zsign + Zpower)2 / delta2 , where SD = 1.1; delta = 0.5; Zsign for 5% type 1 error is 1.96 and Zpower for 20% type 2 error is 0.84, N per group = 76, totalling 152. Inflating our estimate to adjust for attrition/loss to follow-up (conservatively estimated at around 10% at each measurement point), and to account for potential design effects based on sampling within catchment areas (conservatively estimated at 1.1 or an inflation of 10%), our minimum total sample size is 152/0.70 * 1.1 = 240 (120 in each group). A sample of this size is also sufficient to examine mediation effects of at least medium size using the MacKinnon approach [23].

### Randomisation and blinding

Women were asked to complete baseline survey measures prior to randomised group allocation. The statistician created a randomisation sequence using Stata 11.1 (StataCorp, College Station, TX) statistical software, allocating participants to either the intervention or control group in a 1:1 allocation, using random block sizes of 2 and 4. The randomisation schedule was saved in a Microsoft Excel worksheet, which was accessible only by the research fellows responsible for the group allocation of sequential participants, during the allocation period. Except for the statistician and interventionists (dietitians and research fellows administering the intervention), investigators and other staff were kept blind to the allocation of participants. Intervention staff and dietitians who delivered the intervention did not take outcome measurements or analyse data.

### Statistical methods

Descriptive statistics will be used to examine characteristics of the trial participants. Outcomes will be analysed on an intention-to-treat basis using Generalized Estimating Equations to fit regression models describing intervention effects on outcome and mediator variables. Potential confounders (e.g., age, education, country of birth, relationship status, employment status, household income, household composition) will be controlled for as necessary. Mediation analyses will be undertaken using the MacKinnon method [23]. Content and thematic analysis will be used to analyse the qualitative process evaluation data.

## Discussion

The randomised controlled trial of the ShopSmart intervention described in this protocol paper will provide much-needed data on the effects of skills-based intervention approaches for increasing fruit and vegetable consumption amongst a target group at high-risk of inadequate intakes – women of low SEP. This evidence is required in order to form appropriate policy and program responses to the epidemic of obesity and poor nutrition currently facing Australia and other countries globally. If shown to be effective, the approach could be rolled out into scalable skills-based programs that could be mail-, telephone-or web/internet-delivered at relatively low-cost, potentially implemented by governments or health care providers.

The links with Coles supermarkets and the Heart Foundation forged within this project represent rare and invaluable opportunities to work collaboratively to promote healthy eating in partnership with a major national industry player in the grocery retail market, and with a key national NGO committed to improving the eating behaviours and heart health of Australians, particularly those experiencing socioeconomic disadvantage. Other strengths of the study include the strong theoretical basis; application of an intervention mapping approach; use of objectively-assessed purchasing (sales) data; and inclusion of an evaluation of cost-effectiveness, and of unintended consequences.

Recently, a literature review examined whether nutrition interventions are more likely to be effective amongst high- than low-socioeconomic groups [24]. That review found that nutrition interventions were less successful amongst the most socioeconomically disadvantaged participants, potentially leading to a widening of dietary and thus health inequalities. Hence interventions such as ShopSmart 4 Health that focus specifically on addressing the nutrition-related needs of those experiencing socioeconomic disadvantage are urgently required in order to redress inequalities in nutrition and associated health outcomes.

## Abbreviations

READI: Resilience for Eating and Activity Despite Inequality; SEP: Socioeconomic position; SEIFA: Socioeconomic Index for Areas.

## Competing interests

The authors declare that there are no competing interests. Coles supermarkets and the National Heart Foundation of Australia provided in-kind but not financial support for the study.

## Authors’ contributions

KB and DC conceived the study. KB, DC, SAM, HL and VI implemented the study and developed the measures. IL, AG, BM and KB developed the intervention content. KB drafted the manuscript. NA contributed expertise on study design, methods and analysis, conducted sample size calculations, and generated the randomisation sequence. All co-authors contributed to revising the manuscript and all read and approved the final manuscript.

## Pre-publication history

The pre-publication history for this paper can be accessed here:

http://www.biomedcentral.com/1471-2458/13/466/prepub

## References

[B1] Australian Bureau of Statistics 2007–08 National Health Survey: Summary of Results (re-issue) 2009 Canberra: Commonwealth of Australia

[B2] Ball K Crawford D Mishra G Socio-economic inequalities in women’s fruit and vegetable intakes: a multilevel study of individual, social and environmental mediators Public Health Nutr 2006 9 5 623 630 16923294 10.1079/phn2005897

[B3] Giskes K Avendaňo M Brug J Kunst AE A systematic review of studies on socioeconomic inequalities in dietary intakes associated with weight gain and overweight/obesity conducted among European adults Obes Rev 2010 11 6 413 429 19889178 10.1111/j.1467-789X.2009.00658.x

[B4] Lock K Pomerleau J Causer L Altmann D McKee M The global burden of disease attributable to low consumption of fruit and vegetables: implications for the global strategy on diet Bull World Health Organ 2005 83 2 100 108 15744402 PMC2623811

[B5] Inglis V Ball K Crawford D Why do women of low socioeconomic status have poorer dietary behaviours than women of higher socioeconomic status? A qualitative exploration Appetite 2005 45 334 343 10.1016/j.appet.2005.05.003 16171900

[B6] Winkler E Turrell G Confidence to cook vegetables and the buying habits of australian households J Am Diet Assoc 2010 110 5, Supplement S52 S61 10.1016/j.jada.2010.03.007 20399299

[B7] Crawford D Ball K Mishra G Salmon J Timperio A Which food-related behaviours are associated with healthier intakes of fruits and vegetables among women? Public Health Nutr 2007 10 3 256 265 17288623 10.1017/S1368980007246798

[B8] Ammerman AS Lindquist CH Lohr KN Hersey J The efficacy of behavioral interventions to modify dietary fat and fruit and vegetable intake: a review of the evidence Prev Med 2002 35 1 25 41 10.1006/pmed.2002.1028 12079438

[B9] Pomerleau J Lock K Knai C McKee M Interventions designed to increase adult fruit and vegetable intake can be effective: a systematic review of the literature J Nutr 2005 135 10 2486 2495 16177217 10.1093/jn/135.10.2486

[B10] Thomson CA Ravia J A systematic review of behavioral interventions to promote intake of fruit and vegetables J Am Diet Assoc 2011 111 10 1523 1535 10.1016/j.jada.2011.07.013 21963019

[B11] Australian Bureau of Statistics (ABS) Regional population growth, Australia, 2011 Cat. no. 3218.0 2012 Canberra: ABS

[B12] Bittman M Changes at the heart of family households: family responsibilities in Australia 1974–1992 Family Matters 1995 40 1 10 15

[B13] Lake A Hyland R Mathers J Rugg-Gunn A Wood C Adamson A Food shopping and preparation among the 30-somethings: whose job is it? (The ASH30 study) British Food Journal 2006 108 6 475 486 10.1108/00070700610668441

[B14] Australian Government Department of Agriclture, Fisheries and Forestry Australian food Statistics 2010–11 2011 Canberra, Australia: Commonwealth of Australia

[B15] Australian Bureau of Statistics 2033.0.55.001 - Socio-economic Indexes for Areas (SEIFA), Data only, 2006 2008 Canberra: Commonwealth of Australia

[B16] Bandura A Human agency in social cognitive theory Am Psychol 1989 44 9 1175 1184 2782727 10.1037/0003-066x.44.9.1175

[B17] Bartholomew LK Parcel GS Kok G Gottlieb NH Intervention Mapping: Designing Theory and Evidence-Based Health Promotion Programs 2001 Mountain View, CA: McGraw-Hill

[B18] Ball K McNaughton S Ni Mhurchu C Andrianopoulos N Inglis V McNeilly B Le HND Leslie D Pollard C Crawford D Supermarket Healthy Eating for Life (SHELf): protocol of a randomised controlled trial promoting healthy food and beverage consumption through price reduction and skill-building strategies BMC Publ Health 2011 11 1 715 10.1186/1471-2458-11-715 PMC3186753 21936957

[B19] Smith A Kellett E Schmerlaib Y The Australian Guide to Healthy Eating 1998 Canberra: Commonwealth Department of Health and Ageing 22

[B20] Foley RM Pollard CM Food Cent$- implementing and evaluating a nutrition education project focusing on value for money Aust N Z J Public Health 1998 22 494 501 10.1111/j.1467-842X.1998.tb01420.x 9659779

[B21] Australian Bureau of Statistics Australian Bureau of Statistics National Nutrition Survey User’s Guide 1995 1998 Canberra, Australia: Commonwealth of Australia

[B22] Ezzati M Lopez A Rodgers A Murray C Comparative quantification of health risks: global and regional burden of disease attributable to selected major risk factors 2004 Geneva: World Health Organization

[B23] MacKinnon DP Introduction to Statistical Mediation Analysis 2008 NY: Lawrence Erlbaum

[B24] Oldroyd J Burns C Lucas P Haikerwal A Waters E The effectiveness of nutrition interventions on dietary outcomes by relative social disadvantage: a systematic review J Epidemiol Community Health 2008 62 7 573 579 10.1136/jech.2007.066357 18559438

